# Structural correlates of atypical visual and motor cortical oscillations in pediatric‐onset multiple sclerosis

**DOI:** 10.1002/hbm.25126

**Published:** 2020-07-10

**Authors:** Amy T. Waldman, John R. Sollee, Ritobrato Datta, Amy M. Lavery, Geraldine Liu, Tomas S. Aleman, Brenda L. Banwell, William C. Gaetz

**Affiliations:** ^1^ Division of Neurology Children's Hospital of Philadelphia Philadelphia Pennsylvania USA; ^2^ Department of Neurology and Pediatrics Perelman School of Medicine at the University of Pennsylvania Philadelphia Pennsylvania USA; ^3^ Division of Ophthalmology Children's Hospital of Philadelphia Philadelphia Pennsylvania USA; ^4^ Department of Radiology Children's Hospital of Philadelphia Philadelphia Pennsylvania USA

**Keywords:** diffusion tensor imaging, magnetic resonance imaging, MEG, multiple sclerosis, pediatrics, postmovement beta rebound, visual gamma band

## Abstract

We have previously demonstrated that pediatric‐onset multiple sclerosis (POMS) negatively impacts the visual pathway as well as motor processing speed. Relationships between MS‐related diffuse structural damage of gray and white matter (WM) tissue and cortical responses to visual and motor stimuli remain poorly understood. We used magnetoencephalography in 14 POMS patients and 15 age‐ and sex‐matched healthy controls to assess visual gamma (30–80 Hz), motor gamma (60–90 Hz), and motor beta (15–30 Hz) cortical oscillatory responses to a visual‐motor task. Then, 3T MRI was used to: (a) calculate fractional anisotropy (FA) of the posterior visual and corticospinal motor WM pathways and (b) quantify volume and thickness of the cuneus and primary motor cortex. Visual gamma band power was reduced in POMS and was associated with reduced FA of the optic radiations but not with loss of cuneus volume or thickness. Activity in the primary motor cortex, as measured by postmovement beta rebound amplitude associated with peak latency, was decreased in POMS, although this reduction was not predicted by structural metrics. Our findings implicate loss of WM integrity as a contributor to reduced electrical responses in the visual cortex in POMS. Future work in larger cohorts will inform on the cognitive implications of this finding in terms of visual processing function and will determine whether the progressive loss of brain volume known to occur in POMS ultimately contributes to both progressive dysfunction in such tasks as well as progressive reduction in cortical electrical responses in the visual cortex.

## Graphical abstract

We used magnetoencephalography in 14 pediatric‐onset multiple sclerosis (POMS) patients and 15 age‐ and sex‐matched healthy controls to assess visual gamma (30–80 Hz), motor gamma (60–90 Hz), and motor beta (15–30 Hz) cortical oscillatory responses to a visual‐motor task. 3T MRI was used to: (a) calculate fractional anisotropy (FA) of the posterior visual and corticospinal motor WM pathways and (b) quantify volume and thickness of the cuneus and primary motor cortex. Visual gamma band power was reduced in POMS and was associated with reduced FA of the optic radiations but not with loss of cuneus volume or thickness.

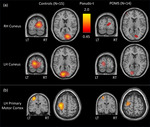

## 1 INTRODUCTION

Multiple sclerosis (MS) is characterized by focal and more subtle diffuse demyelination as well as axonal and neuronal loss, leading to both episodic and ultimately progressive neurological impairment. Damage to the visual pathway is a common manifestation of the disease, and as such, the visual system provides a model for studying the relative contributions of white matter (WM) and gray matter (GM) injury to visual function. MS leads to retrograde degeneration of the retinal nerve fiber layer (RNFL) resulting from acute inflammatory damage of the optic nerve (i.e., optic neuritis [ON]) and also leads to anterograde and retrograde degeneration between the retina and visual cortex, even in the absence of ON (Balk et al., 2015; Gabilondo et al., 2014; Kolasinski et al., 2012).

MS is a chronic disease, with accumulation of tissue injury over time. The onset of MS during childhood or adolescence (pediatric‐onset MS [POMS]) is associated with a relapsing–remitting MS (RRMS) disease course that is very similar to RRMS in adults, with a few key distinctions (Simone et al., 2002): (a) POMS is associated with a higher relapse rate early in the disease as compared to adults, indicative of a more inflammatory disease state (Gorman, Healy, Polgar‐Turcsanyi, & Chitnis, 2009); (b) the young age of POMS patients precludes secondary comorbid disease contributions to brain injury (Marrie et al., 2015), allowing disruption of brain tissue and function to be more directly attributable to MS; and (c) analysis in these young MS patients clearly avoids confounds of prolonged disease. In aggregate, it can be argued that study of POMS provides a view of the earliest impacts of MS pathobiology.

Our previous work has focused on defining and quantifying the extent of axonal and neuronal loss of the visual system in POMS and understanding the subsequent functional outcomes in children (Datta et al., 2019; Waldman, Ghezzi, et al., 2014; Waldman, Hiremath, et al., 2014). As in adults with MS, retrograde RNFL damage from acute ON has been demonstrated in POMS (Graves et al., 2017; Waldman et al., 2017; Waldman, Ghezzi, et al., 2014; Waldman, Hiremath, et al., 2014). Interestingly, following acute ON in children, only about 50% of affected eyes demonstrate RNFL thinning, suggestive of some measure of resilience or greater reparative capacity in children relative to adult MS patients (Waldman et al., 2017; Waldman, Ghezzi, et al., 2014; Waldman, Hiremath, et al., 2014; Yeh et al., 2009). In our recent work, we demonstrated thinning of the visual cortex in 20 POMS subjects compared to 22 age‐ and sex‐matched healthy controls; however, cortical thinning was not associated with RNFL thinning or a history of ON (Datta et al., 2019). Thus, we were unable to replicate the adult MS findings of trans‐synaptic anterograde and retrograde degeneration between the retina and the cortex. Of note, however, we detected reduced mean fractional anisotropy (FA) using diffusion tensor imaging (DTI) of the optic radiations in POMS patients which persisted in normal‐appearing WM after T2 hyperintense lesions were removed (Datta et al., 2019). Furthermore, there was an association between visual cortical mantle thinning and decreased mean FA of the optic radiations in POMS patients (Datta et al., 2019). Based on these findings, we postulate that loss of WM integrity is an early facet of POMS, and disruption of WM tracts may contribute to anterograde loss of cortical tissue. However, the consequences of such damage on cortical electrophysiological activity remain unclear.

We used magnetoencephalography (MEG), paired with structural MRI and DTI, to explore the cortical responses generated by peripherally presented visual stimuli in POMS and their relationship to the integrity of WM and GM in the visual pathway. MEG is a noninvasive imaging modality that directly measures neuronal oscillations in the cortex with high temporal (millisecond) resolution (Hämäläinen, Hari, Ilmoniemi, Knuutila, & Lounasmaa, 1993). When accompanied by a structural MRI, high spatial resolution is also achieved (Hämäläinen et al., 1993). Increases in oscillatory activity within the gamma band (30–80 Hz) are signatures of information processing within the associated cortical regions (Muthukumaraswamy & Singh, 2013). Gamma band activity has been implicated in attention (Fries, Reynolds, Rorie, & Desimone, 2001; Fries, Schröder, Roelfsema, Singer, & Engel, 2002), memory (Jensen, Kaiser, & Lachaux, 2007) visual perception (Adjamian et al., 2004; Hall et al., 2005; Melloni et al., 2007; Parra et al., 2003), object recognition (Cheyne, Bells, Ferrari, Gaetz, & Bostan, 2008), and motor control (Cheyne et al., 2008; Muthukumaraswamy, 2010). Visual cortex gamma oscillations, elicited by simple visual stimuli patterns such as square wave gratings, have been demonstrated in several prior studies, including those with peripherally presented stimuli (Gaetz, Roberts, Singh, & Muthukumaraswamy, 2012; Muthukumaraswamy, Edden, Jones, Swettenham, & Singh, 2009). These oscillations have been shown to be highly stable and reproducible within subjects (Muthukumaraswamy, Singh, Swettenham, & Jones, 2010). In addition to measuring visual cortex responses, MEG has also been used to assess oscillatory changes in the motor beta (15–30 Hz) (Arpin et al., 2017) and motor gamma (60–90 Hz) bands (Cheyne & Ferrari, 2013) of the motor cortex.

Building upon our prior observations of visual cortex thinning and disruption of subserving WM pathways, we studied the interplay between structural and electrophysiologic responses to visual stimuli. We hypothesized that the visual gamma and motor beta and gamma band responses are altered in POMS compared to healthy youth and are related to the degree of structural injury along the visual and motor pathways. To test these hypotheses, we presented POMS subjects and healthy controls with a visual stimulus while recording cortical oscillations with MEG. A button press was incorporated into each trial to ensure attention and to elicit activity in the motor cortex. We then compared visual gamma and motor gamma and beta oscillations between POMS and healthy controls and investigated their relationships to the WM and cortical integrity of relevant tracts and regions.

## 2 METHODS

### 2.1 Participants

Participants with relapsing–remitting POMS, as defined by the 2017 McDonald criteria (Thompson et al., 2018) and whose first attack occurred at less than 18 years of age, were recruited from the Pediatric MS Program at the Children's Hospital of Philadelphia (CHOP). Medical records for POMS participants were reviewed to determine history of ON. ON was defined as visual impairment lasting more than 24 hr accompanied by a change in color vision or visual fields and the presence of pain with eye movements. Patients were excluded if they had an episode of ON or received corticosteroid treatment within the last 6 months. Participants were permitted to wear contact lenses if required. Healthy youth with normal corrected visual acuity (20/25 acuity or better) and no ophthalmologic or neurologic diseases were recruited by local advertisement. The study was approved by the CHOP Institutional Review Board, all participants gave informed written consent, and child assent was obtained.

### 2.2 Clinical measures

History of ON and annualized relapse rate were recorded for POMS patients and confirmed by medical record review. Expanded Disability Status Scale (EDSS) scores were assessed for POMS patients at the time of data collection.

### 2.3 Task paradigm

Visual stimuli used in this study are identical to those reported previously (Gaetz et al., 2012; Muthukumaraswamy et al., 2009). Briefly, visual stimuli consisted of vertical, stationary, 100% contrast, black and white square‐wave gratings (3 cycles per degree, mean luminance) presented separately to lower left and right of fixation. The stimulus presented to the left of fixation was subtended 4° both horizontally and vertically with the upper right corner of the stimulus located 0.5° from a small, red centrally presented fixation cross (Gaetz et al., 2012; Muthukumaraswamy et al., 2009). The stimulus presented to the right of fixation was presented with the upper left corner equidistant to the fixation cross as the opposite side. It has been demonstrated that high contrast stimuli (Hall et al., 2005; Henrie & Shapley, 2005) with a spatial frequency of 3 cycles per degree maximize gamma oscillations (Adjamian et al., 2004); furthermore, square wave stimuli tend to produce greater gamma activity than sine wave stimuli (Muthukumaraswamy & Singh, 2009).

In order to ensure that participants attended to each visually presented stimulus, participants were instructed to maintain fixation on the centrally presented red cross throughout the experiment and to use their right index finger to press a button at the disappearance of the stimulus. In order to prevent anticipatory button presses, the length of stimulus presentation varied by trial (minimum 1.5 s to 2 s maximum, average duration of 1.75 s). Stimuli were presented in a block of 100 continuously presented trials. Subjects were required to respond within 700 ms in order for the next stimulus to occur. Failure to respond in time resulted in a prompt that the response was “too slow.” All stimulus presentations were controlled by Presentation software (Version 18.0, Neurobehavioral Systems, Inc., Berkeley, CA, www.neurobs.com).

### 2.4 MEG data acquisition

Whole‐head MEG recordings were conducted using a CTF‐Omega 275 channel system (CTF MEG International Services). Whole‐head recordings were sampled at 1,200 Hz (0–300 Hz bandpass) and acquired with participants in the seated upright position. Prior to data acquisition, three localization fiducial coils were placed at the nasion and preauricular locations and used for coregistration with the subject's brain MRI.

While all the POMS subjects were tested using the same projector (Sanyo Protrax Multiverse), 9 of the 15 healthy participants (enrolled later in the study to age and sex match) were tested on upgraded projector hardware (NEC, High Definition Multimedia Interface, model PA521U).

### 2.5 MEG data analysis

To assess visual gamma band responses, the continuously recorded data were first epoched (4 s duration; 2 s pre 2 s post) with the visual stimulus onset as time‐zero. For analysis of the motor response, the continuously recorded data were reepoched around the button‐press response (4 s duration; 2 s pre 2 s post recording). Trials with more than 1 cm of head motion were excluded. To remove trials with excessive noise, all epoched MEG data were filtered using a 1–100 Hz band‐pass filter with a third order gradient applied and direct current offset removed (based on whole trial). This was performed using the vendor provided software, DataEditor version 5.3.

The Synthetic Aperture Magnetometry (SAM) beamformer algorithm was used for source localization. Previously published findings (Cheyne et al., 2008) have shown that very large (>100 pT) artifacts due to dental hardware (i.e., braces) can be successfully addressed using beamformer spatial filter methods. In such cases, the beamformer projects out sources of correlated noise due to these artifacts (and others). In the current study, 1 control had a permanent retainer wire, thus we set a trial rejection limit of 10 pT (root mean square) to exclude noise sources too large to originate from nonbiological sources in an effort to allow covariance performance to be uniform over subjects.

**FIGURE 1:**
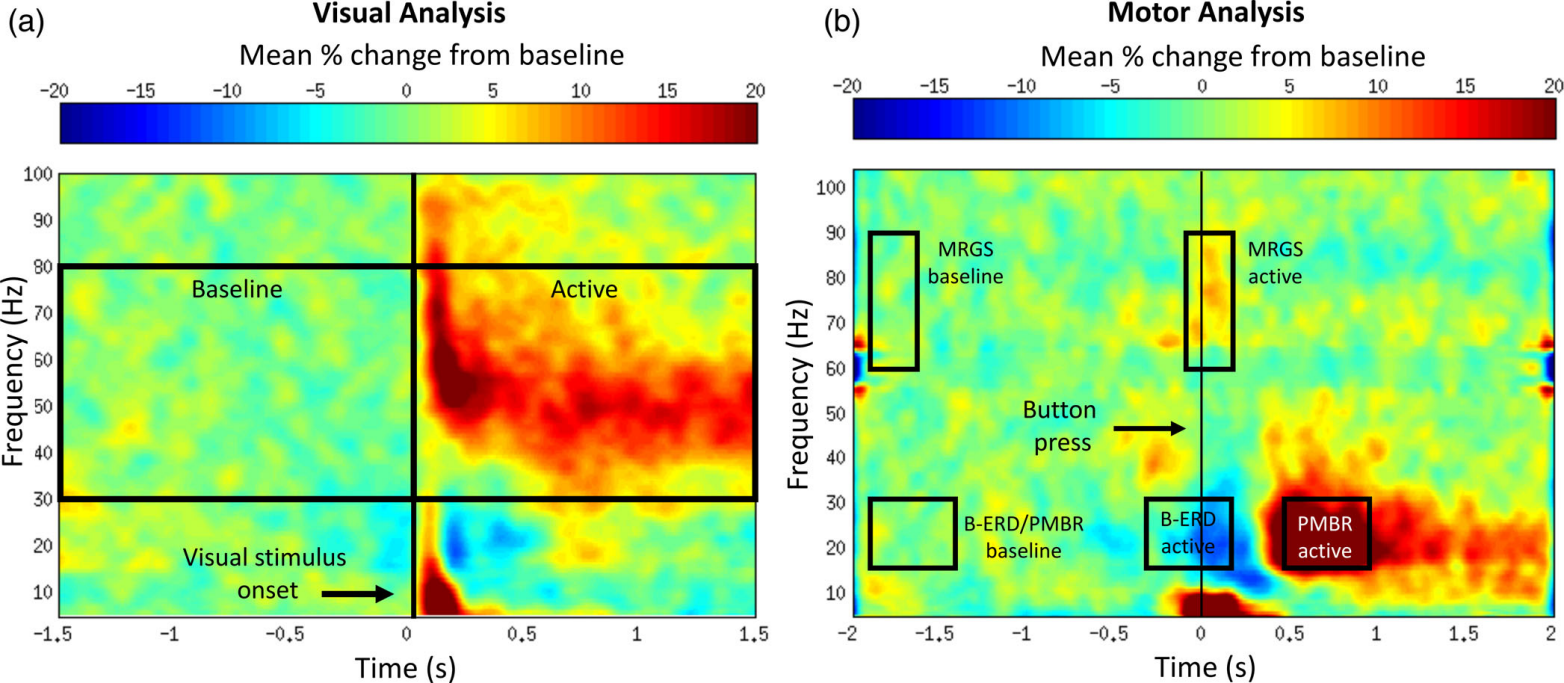
Baseline and active time windows used for differential source activity analysis. LH, left hemisphere; LT, left temporal; POMS, pediatric‐onset multiple sclerosis; RH, right hemisphere; RT, right temporal. (a) Differential source power in the visual gamma band (30–80 Hz) was assessed using a 1,500 ms active window (0.0–1.5 s) relative to a 1,500 ms baseline window (−1.5 to 0.0 s). Time zero (0.0 s) on the x‐axis represents the onset of the visual stimulus. (b) Differential source activity in the beta band (15–30 Hz) (i.e., beta band event‐related desynchrony [B‐ERD]) was assessed using a 500 ms active window (−0.3 to 0.2 s) relative to a 500 ms baseline window (−1.8 to −1.3 s). The expected synchronization of beta band (15–30 Hz) power following transient movements (i.e., postmovement beta rebound [PMBR]) was assessed using a 500 ms active window (0.5–1.0 s) relative to the same baseline window (−1.8 to −1.3 s). Movement‐related gamma band (60–90 Hz) synchrony (MRGS) was assessed using a 300 ms active window (−0.1 to 0.2 s) relative to a 300 ms (−1.8 to −1.5 s) baseline window. Time zero on the x‐axis (0.0 s) represented the button press

For each subject, noise‐normalized differential power values were calculated (integrated across a spectro‐temporal “active” window compared to a “baseline” window) at the location of each individual's peak response and expressed as the pseudo‐t statistic, hereafter abbreviated as “pseudo‐t” (Nichols & Holmes, 2001). Here, SAM results are reported as increases or decreases of noise‐normalized differential source power in units of pseudo‐t (hereafter referred to as visual gamma power). Using SAM, we assessed stimulus‐locked increases associated with the visual gamma band (30–80 Hz) response by measuring SAM differential source activity using 1,500 ms time windows placed at 0.0–1.5 s (active) and −1.5 to 0.0 s (baseline) (stimulus onset = 0.0 s). These baseline and active windows were chosen in accordance with previous work performed by one of the authors (W. C. G.) investigating primary visual cortex (V1) gamma band responses in healthy development (Gaetz et al., 2012). A schematic of the baseline and active windows chosen for the visual analysis are depicted in Figure 1a.

To explore group differences in motor cortical oscillations associated with the button‐press response, differential source activity in the beta band (15–30 Hz) (i.e., beta band event‐related desynchrony; B‐ERD) was assessed using a 500 ms active window (−0.3 to 0.2 s) relative to a 500 ms premovement baseline time period locked to the button press response (−1.8 to −1.3 s). To assess the expected synchronization of beta band power following transient movements (i.e., postmovement beta rebound; PMBR) a 0.5–1.0 s active time window was compared to the same premovement baseline (−1.8 to −1.3 s). In addition, movement‐related gamma band synchrony (MRGS; 60–90 Hz) was assessed using a 300 ms active window (−0.1 to 0.2 s) relative to the button press and referenced to a 300 ms (−1.8 to −1.5 s) premovement baseline period. These baseline and active windows are consistent with those chosen in previous MEG work on the motor system (Gaetz, Edgar, Wang, & Roberts, 2011; Gaetz et al., 2019). A schematic of the baseline and active windows chosen for the motor analysis is depicted in Figure 1b.

The reaction time (amount of time elapsed between the stimulus disappearance and the button‐press response) was recorded for each subject. All button presses that occurred <200 ms from stimulus offset were assumed to be anticipatory trials and were thus manually removed prior to data analysis.

### 2.6 MEG group analysis

MRI structural images and the individual differential SAM beamformer results for visual gamma band power, B‐ERD, MRGS, and PMBR were first normalized to the Montreal Neurologic Institute (MNI) 152 template using a nonlinear transform (FNIRT; Andersson, Jenkinson, & Smith, 2007) and averaged separately within group and frequency band of interest. Observed group‐averaged peak MNI locations and associated Talairach labels were determined.

### 2.7 Region of interest time–frequency analysis

The beamformer weights vector for the group‐level peak location was explicitly computed (e.g., for visual gamma band analysis the covariance calculated from −2 to 2 s; 30–80 Hz) and subsequently, the sensor data were projected through these weights to obtain a time‐varying estimate of the activity at the group‐averaged image peak location. Time–frequency responses (TFRs) for group‐level peak locations were evaluated by transforming the region of interest (ROI) coordinates (observed in MNI space) back into each individual's (CTF) MRI coordinate system. These group‐level peak locations were then used to assess virtual sensor TFR analysis using the Hilbert transform. TFR analyses of source waveforms for each individual's ROI locations were conducted at 0.5 Hz frequency steps between 1 and 100 Hz and represented as a percentage change from baseline for each frequency band of interest.

### 2.8 PMBR time‐to‐peak analysis

PMBR time‐to‐peak latency was measured as the latency associated with the maximum PMBR amplitude observed from the filtered (15–30 Hz) virtual sensor source waveform (i.e., at the group‐level peak PMBR location). For each POMS and healthy control subject, we averaged the PMBR virtual sensor time course and noted the latency corresponding to the maximum PMBR amplitude. These PMBR source waveforms were then averaged within group to compare group level differences in PMBR time‐to‐peak amplitude.

### 2.9 MRI and DTI acquisition

The MRI data were acquired on the same Siemens Verio 3T scanner with a 32‐channel head coil. Anatomical images were acquired using T1‐weighted high‐resolution anatomical scan using Fast Low Angle Shot (FLASH) (192 slices, 1 mm isotropic, repetition time [TR] = 20 ms, echo time [TE] = 5 ms, field of view [FOV] = 256 mm, flip‐angle 27) and 3D FLAIR (208 slices, 1 mm isotropic, TR = 5,000 ms; TE = 388 ms; FOV = 256 mm) acquisitions. 64‐direction DTI was collected using a 2D echo‐planar imaging sequence with a *b*‐value of 1,000 s/mm^2^ (TR = 10,300 ms; TE = 95 ms; FOV = 256 mm; number of slices = 50; voxel size = 2.0 mm isotropic).

### 2.10 MRI data analyses

All images were visually inspected to ensure the images lacked motion artifact and signal dropouts. T1 FLASH images were processed with FreeSurfer toolkit (https://surfer.nmr.mgh.harvard.edu, version 6). Lesion Segmentation Toolbox (Schmidt et al., 2012) was used to segment T2 hyperintense lesions from a combination of T1 and FLAIR images. The pipeline first segments the T1 image into the three main tissue classes (cerebrospinal fluid [CSF], GM, and WM). This information is then combined with the FLAIR intensities in order to calculate lesion probability maps. Almost all WM lesions in the T1 FLASH images were automatically filled in the WM hypointensity filling step in the default FreeSurfer pipeline, similar to findings reported by others (Guo, Ferreira, Fink, Westman, & Granberg, 2019). To ensure that no WM lesions were unaccounted for, manual inspection and filling of the lesions (if required) were performed on the output of the WM hypointensity filling step. The rest of the FreeSurfer pipeline was run following this step. After pial (GM/CSF, that is, outer boundary of cortical mantle) and GM/WM (i.e., inner boundary of cortical mantle) surfaces were reconstructed (Datta et al., 2019), quality check was performed on each subject's processed data by manually examining each slice to ensure accurate GM and WM segmentation. For regions with segmentation errors, control points were added manually to indicate WM voxels that were erroneously classified as GM, as described previously (Datta et al., 2019). The pipeline was rerun, and the data were rechecked to again ensure segmentation accuracy. Cortical thickness was estimated at each point across the cortical mantle by calculating the distance between the GM/WM boundary and the pial boundary.

Previously published findings involving healthy children as well as adults with MS showed that the visual gamma band response to a stimulus presented laterally to a central fixation cross typically produces a peak response in the contralateral cuneus (Barratt et al., 2017; Gaetz et al., 2012; Muthukumaraswamy et al., 2009). Thus, for each subject, we used the cuneus in the hemisphere contralateral to the visual stimulus presentation as the ROI for volume and thickness measures. The primary motor cortex was chosen as the ROI for the motor analysis, as motor beta activity has been shown to reach peak amplitudes in this region (Gaetz et al., 2011; Gaetz et al., 2019; Little, Bonaiuto, Barnes, & Bestmann, 2019). Desikan Atlas was used to define the cuneus and primary motor cortex (Desikan et al., 2006), and FreeSurfer was utilized to determine the volume and thickness of these regions. Regional volumes were adjusted (normalized) for head size using the proportion method (tissue‐to‐intracranial volume ratio) (Obenaus, Yong‐Hing, Tong, & Sarty, 2001; Sanfilipo, Benedict, Zivadinov, & Bakshi, 2004; Voevodskaya, 2014). Total intracranial volume was estimated in FreeSurfer.

### 2.11 DTI data analyses

The Tract‐Based Spatial Statistics (Smith et al., 2006) processing pipeline was used to align DTI data. For each subject, an FA image was created by fitting a tensor model to the raw diffusion data using the Diffusion Toolbox (FDT) available in FMRIB Software Library (FSL—www.fmrib.ox.ac.uk/fsl/). Each subject's FA images was then aligned to every other FA image using nonlinear transformation. The “most representative” FA image was automatically identified as the one that required the smallest amount of average warping when used as an alignment target. The chosen FA image target was then affine‐aligned into MNI 152 standard space, and every FA image was transformed in 1 mm isotropic MNI space by combining the nonlinear transform to the target FA image with the affine transform from that target to MNI space. Next, the mean FA image in MNI space was created and thinned to create a mean FA skeleton, which represents the centers of all tracts common to the group (Smith et al., 2006). Each subject's aligned FA data were then projected onto this skeleton, and mean FA values for the corticospinal tracts and optic radiations, as outlined in the Julich Histological Atlas (Eickhoff et al., 2005), were extracted for each subject.

### 2.12 Statistical analysis

Demographics were compared between the POMS and control groups using Student's *t* test for age and test of proportions for sex. Generalized estimating equations (GEE, using an independent covariance matrix) were used to compare visual gamma power, optic radiation FA, and cuneus volume and thickness between groups, since each subject had two values (one value per hemisphere). Linear regression models were used to compare primary motor cortex oscillation activity (B‐ERD, MRGS, PMBR), primary motor cortex volume and thickness, and corticospinal tract FA between groups, since the right‐hand button press was tested for left hemisphere motor cortical responses only (one value per subject). All models for group‐level comparisons included age at scan as an adjustment variable. ON history was also checked for confounding in our primary analysis (between visual gamma power in POMS vs. controls).

For MEG variables that significantly differed between the POMS and control groups (*p* value <.05), their relationships to structural measures (cortical volume and thickness and FA) were investigated in the entire cohort (both POMS and healthy controls) using multivariate GEE models or linear regression, where appropriate, with disease group (POMS vs. control) and age as adjustment factors. All analyses were performed using Stata Statistical Software (STATA, Version 12.1, College Station, TX: StataCorp LP); Statistical Analysis Software (SAS, Version 8, Cary, NC: SAS Institute Inc.) was used to generate predicted values from the GEE models, and plots were generated using GraphPad Prism (Version 8.3.0, La Jolla, CA, GraphPad Software Inc.).

## 3 RESULTS

### 3.1 Clinical findings

POMS subjects (*N* = 14) and healthy controls (*N* = 15) were enrolled (Table 1). Controls were age and sex matched when possible: nine POMS subjects were sex‐ and age‐matched within 1 year, while four POMS subjects were age‐matched within 3 years. There were no differences in age or sex between POMS and controls (Table 1). Seven POMS patients had a remote history of ON, five with unilateral and two with bilateral ON. Additional clinical features for the POMS group are presented in Table 1. The POMS group had an average disease duration of 3.14 years, median EDSS of 1, and a mean annualized relapse rate of 1.69.

**TABLE 1:** Demographics and clinical features

	Controls (*N* = 15)	POMS (*N* = 14)	*p*‐Value^a^
Age (years), mean (*SD*, range)	18.7 (2.0, 15.9–23.6)	17.5 (3.1, 11.0–24.4)	0.22
Sex, *N* (% female)	10 (66.7)	6 (42.9)	0.20
History of ON, *N* (%)	N/A	7 (50)	N/A
Disease duration (years), mean (*SD*, range)	N/A	3.14 (3.06, 0.27–11.77)	N/A
EDSS, median (range)	N/A	1 (0–3.5)	N/A
Annualized relapse rate, mean (*SD*, range)	N/A	1.69 (1.2, 0.25–3.63)	N/A

Abbreviations: EDSS, Expanded Disability Status Scale; N/A, not applicable; ON, optic neuritis; POMS, pediatric‐onset multiple sclerosis; *SD*, standard deviation.

^a^ Age was compared using the Student's *t* test for independent samples. The proportion of females to males was compared using the test of proportions.

### 3.2 Visual gamma band response

After dropping trials for excessive head motion and noise (>10 pT), an average of 92.8 (*SD* 5.6) trials for controls and 90.7 (*SD* 4.1) trials for POMS subjects were retained and included in the visual gamma band differential source power analysis. The number of retained trials did not differ between controls and POMS subjects.

A visual gamma band peak response was detected in 23 of the 28 contralateral hemispheres in the POMS group compared to 28 of 30 in the control group (Table 2). Only one subject, a POMS patient, did not produce any visual gamma response (change in power relative to baseline) to left‐ or right‐hemifield presented visual stimuli. Two POMS subjects produced contralateral peak visual gamma responses (right hemisphere) to left‐hemifield presented visual stimuli but did not produce contralateral (left hemisphere) responses to right‐hemifield presented visual stimuli. One POMS subject produced a peak visual gamma response in the hemisphere ipsilateral (left hemisphere) to the hemifield of visual stimulus presentation (left hemifield). For purposes of the visual gamma analysis, this single ipsilateral response was treated akin to a complete absence of a response. Visual gamma power was assigned a value of zero pseudo‐t if the response was not detected in the contralateral hemisphere.

**TABLE 2:** Visual gamma responses and power for each subject

Group	Age (years)	Sex	ON history	Visual stimulus presented in lower left hemifield		Visual stimulus presented in lower right hemifield	
				Visual gamma response (location)	Visual gamma power, pseudo‐t	Visual gamma response (location)	Visual gamma power, pseudo‐t
POMS	19.78	M	Left	Present, RH	1.6	Present, LH	1.6
POMS	17.75	M	None	Absent	—	Absent	—
POMS	24.39	M	Bilateral	Ipsilateral, LH	—	Present, LH	0.5
POMS	13.36	F	Left	Present, RH	2.1	Present, LH	0.8
POMS	18.62	M	None	Present, RH	1.9	Present, LH	1.5
POMS	14.83	M	Left	Present, RH	0.3	Present, LH	1.6
POMS	16.73	F	None	Present, RH	0.7	Present, LH	0.6
POMS	18.16	F	None	Present, RH	1.3	Present, LH	0.6
POMS	19.30	M	None	Absent	—	Present, LH	0.6
POMS	11.00	F	Right	Present, RH	0.5	Present, LH	0.7
POMS	18.02	M	None	Present, RH	1.7	Present, LH	2.9
POMS	17.89	F	Bilateral	Absent	—	Present, LH	0.4
POMS	17.07	F	Left	Present, RH	1.6	Present, LH	2.7
POMS	18.51	M	None	Present, RH	0.9	Present, LH	2.2
Control	18.76	F	N/A	Present, RH	4.5	Present, LH	3.1
Control	20.00	F	N/A	Present, RH	2.8	Present, LH	3.6
Control	16.15	F	N/A	Present, RH	1.6	Present, LH	1.9
Control	18.47	F	N/A	Present, RH	0.7	Present, LH	1.9
Control	16.60	F	N/A	Present, RH	0.9	Present, LH	2.0
Control	23.58	F	N/A	Present, RH	1.7	Present, LH	3.4
Control	15.87	F	N/A	Present, RH	2.5	Present, LH	2.5
Control	20.36	F	N/A	Absent	—	Present, LH	1.6
Control	19.92	F	N/A	Present, RH	0.6	Present, LH	0.3
Control	17.31	F	N/A	Present, RH	0.7	Present, LH	2.2
Control	17.48	M	N/A	Present, RH	2.8	Present, LH	2.6
Control	17.62	M	N/A	Present, RH	1.4	Present, LH	0.5
Control	20.02	M	N/A	Absent	—	Present, LH	2.4
Control	19.65	M	N/A	Present, RH	6.5	Present, LH	5.5
Control	19.24	M	N/A	Present, RH	2.3	Present, LH	2.8

Abbreviations: F, female; M, male; N/A, not applicable; ON, optic neuritis; LH, left hemisphere of the brain; POMS, pediatric‐onset multiple sclerosis; RH, right hemisphere of the brain.

**FIGURE 2:**
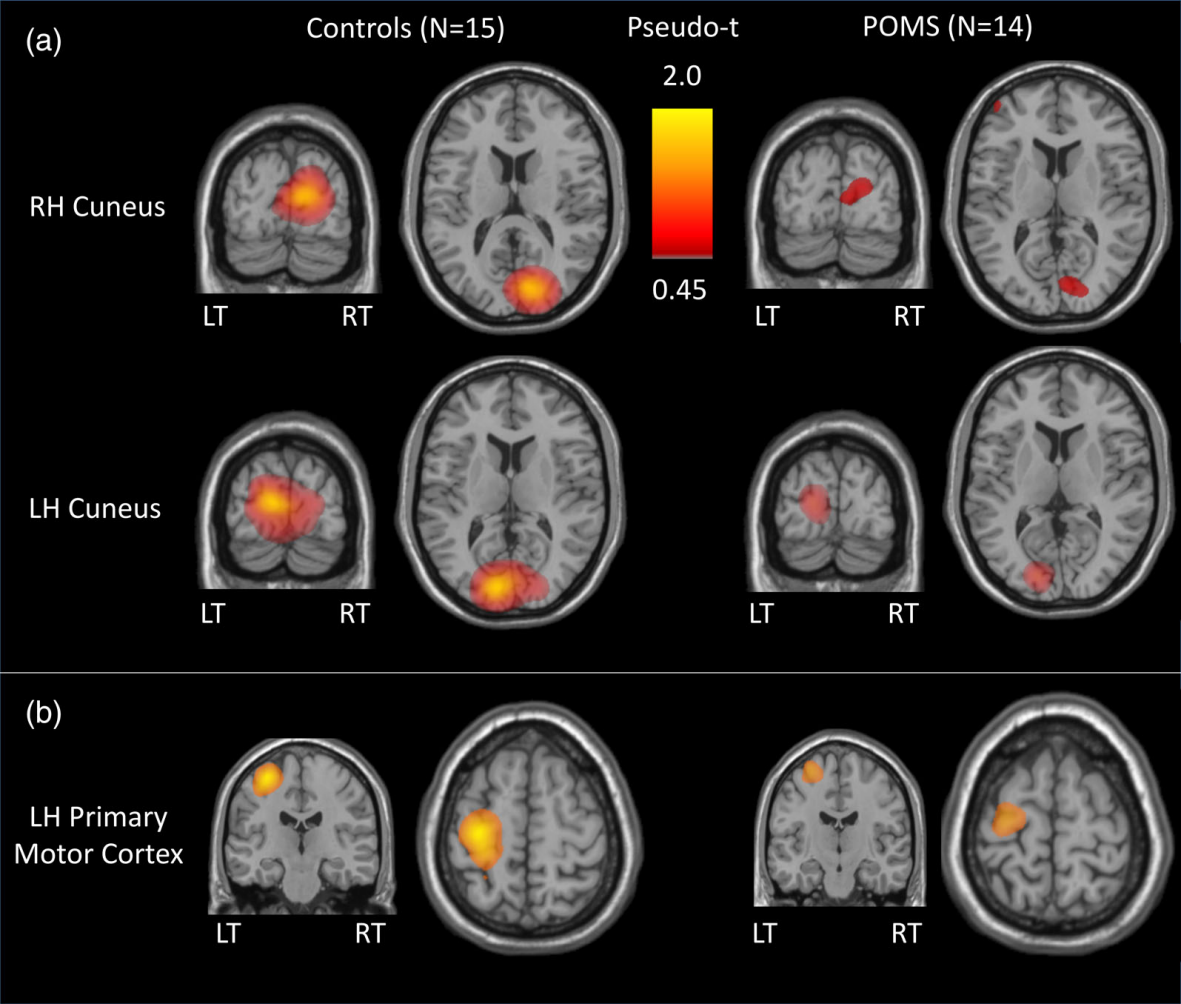
Visual gamma band and postmovement beta rebound cortical differential activity. LH, left hemisphere; LT, left temporal; POMS, pediatric‐onset multiple sclerosis; RH, right hemisphere; RT, right temporal. (a) The cortical response to left and right visual gamma stimuli is compared in controls and POMS subjects. For stimuli presented in the lower‐left hemifield, the group‐averaged location of peak visual gamma power is observed in the right hemisphere cuneus at Talairach coordinates (11, −83, 15) for controls and (9, −83, 11) for POMS subjects. For stimuli presented in the lower‐right hemifield, the group‐averaged location of peak visual gamma power is observed in the left hemisphere cuneus at Talairach coordinates (−11, −87, 11) for controls and (−17, −87, 9) for POMS subjects. Visual gamma power is reduced in POMS subjects compared to controls. (b) The group‐averaged PMBR response observed following right finger button press is compared for controls and POMS patients. The group‐averaged location of peak PMBR response is observed in the left primary motor cortex at Talairach coordinates (−33, −21, 61) for controls and (−29, −15, 67) for POMS subjects

**FIGURE 3:**
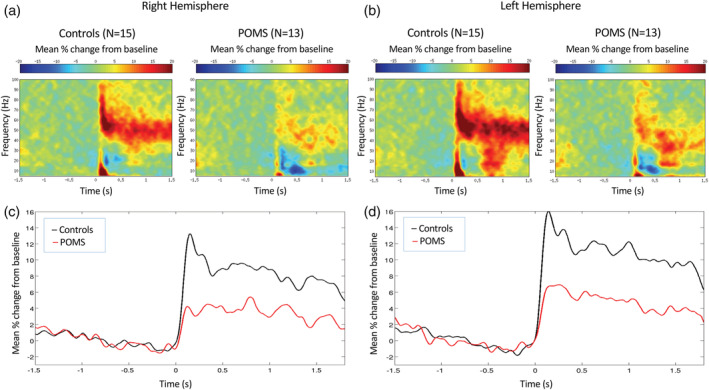
Upper plots (a,b): Group‐averaged time‐frequency plots are shown for control and POMS. Results show reduced visual gamma band activity observed from both the right (a) and left (b) hemispheres. Lower plots (c,d): Visual gamma power (averaged over the 30–80 Hz) is contrasted between POMS (red) and control (black) responses from right (c) and left (d) hemispheres. POMS, pediatric‐onset multiple sclerosis; s, seconds

Visual gamma power was reduced in the POMS group (mean 1.05, *SD* 0.84) relative to controls (mean 2.18, *SD* 1.52, *p* = .001, Table 3, Figure 2a). Visual gamma band TFR plots and mean visual gamma amplitude plots are depicted in Figure 3. We conducted a sensitivity analysis removing the zero pseudo‐t power values for those subjects for whom a visual gamma response was not detected (*N* = 5) or for whom a peak response was detected in the hemisphere ipsilateral to the stimuli (*N* = 1), as assigning values of zero pseudo‐t could bias the POMS group mean. After restricting the analysis, the group‐level power differences remained (POMS mean 1.27, *SD* 0.76 vs. control mean 2.3, *SD* 1.5; beta coefficient = .95; 95% confidence interval = −1.61, −0.28; *p* = .005).

**TABLE 3:** Cortical oscillatory, structural, and behavioral differences between controls and POMS subjects

	Controls mean (*SD*, range)	POMS mean (*SD*, range)	Unadjusted models^a^		Adjusted models^b^	
			Beta coefficient (95% CI)	*p*‐Value	Beta coefficient (95% CI)	*p*‐Value
*MEG: Visual gamma*						
Power (pseudo‐t)	2.18 (1.52, 0–6.50)	1.05 (0.84, 0–2.90)	−1.13 (−1.76, −0.50)	**<.001**	−1.12 (−1.76, −0.47)	**.001**
*MEG: Motor gamma (left hemisphere only)*						
MRGS (pseudo‐t)	2.09 (1.22, 0.80–4.10)	1.50 (0.96, 0.25–3.50)	−0.60 (−1.52, 0.31)	.184	−0.60 (−1.54, 0.34)	.201
B‐ERD (pseudo‐t)	−2.30 (1.05, −0.40 – −4.80)	−2.43 (0.57, −1.8 – −3.2)	0.13 (−0.55, 0.81)	.694	0.18 (−0.51, 0.87)	.595
PMBR time‐to‐peak latency (ms)	665.67 (158.44, 418.00–888.90)	710.32 (153.63, 458.00–979.00)	44.64 (−77.05, 166.34)	.458	36.39 (−87.66, 160.43)	.551
PMBR amplitude associated with peak latency (% change from baseline)	38.85 (25.77, 9.54–98.03)	17.46 (11.72, 3.62–42.21)	−21.40 (−37.38, −5.41)	**.011**	−20.67 (−37.09, −4.24)	**.016**
*MRI: Cuneus*						
Normalized volume	1.76E‐3 (0.33E‐3, 1.27E‐3–2.63E‐3)	1.52E‐3 (0.29E‐3, 0.88E‐3–2.09E‐3)	−0.24E‐3 (−0.40E‐3, −0.08E‐3)	**.003**	−0.24E‐3 (−0.40E‐33, −0.08E‐3)	**.003**
Thickness (mm)	1.80 (0.16, 1.56–2.23)	1.74 (0.15, 1.52–2.10)	−0.06 (−0.14, 0.02)	.117	−0.07 (−0.15, 0.01)	.101
*MRI: Primary motor cortex (left hemisphere only)*						
Normalized volume	6.00E‐3 (0.72E‐3, 5.01E‐3–7.31E‐3)	5.57E‐3 (0.61E‐3, 4.68E‐3–6.68E‐3)	−0.43E‐3 (−0.95E‐3, 0.09E‐3)	.102	−0.43E‐3 (−0.97E‐3, 0.11E‐3)	.117
Thickness (mm)	4.50 (0.45, 3.60–5.24)	4.31 (0.29, 3.90–4.77)	−0.19 (−0.49, 0.11)	.205	−0.17 (−0.48, 0.13)	.261
*DTI: Optic radiations*						
FA	0.50 (0.03, 0.44–0.55)	0.46 (0.04, 0.37–0.54)	−0.03 (−0.05, −0.02)	**<.001**	−0.04 (−0.05, −0.02)	**<.001**
*DTI: Corticospinal tract (left hemisphere only)*						
FA	0.54 (0.04, 0.49–0.66)	0.53 (0.03, 0.48–0.57)	−0.01 (−0.04, 0.02)	.463	−0.01 (−0.03, 0.02)	.597
*Reaction time*						
RT (ms)	347.43 (64.29, 271.01–473.61)	416.75 (69.84, 283.20–510.60)	69.32 (17.20, 121.43)	**.011**	63.00 (11.69, 114.32)	**.018**

*Note:* All statistically significant values are provided in bold.

Abbreviations: B‐ERD, beta event‐related desynchrony; DTI, diffusion tensor imaging; FA, fractional anisotropy; MEG, magnetoencephalography; mm, millimeters; MRGS, movement‐related gamma synchrony; ms, milliseconds; PMBR, postmovement beta rebound; POMS, pediatric‐onset multiple sclerosis; RT, reaction time.

^a^ Univariate models exploring differences between POMS subjects and healthy controls.

^b^ Multivariate models exploring differences between POMS subjects and healthy controls, adjusted for age.

Of note, a history of ON did not influence the presence or absence of gamma band responses (Table 2) or mean visual gamma power (beta coefficient = −.22; 95% confidence interval = −1.2, 0.75; *p* = .653). Visual gamma power in the control group was not significantly affected by the mid‐study upgrade in projector hardware (beta coefficient = −.275; *p* = .620; 95% CI −1.36, 0.81).

### 3.3 DTI of the optic radiations

**FIGURE 4:**
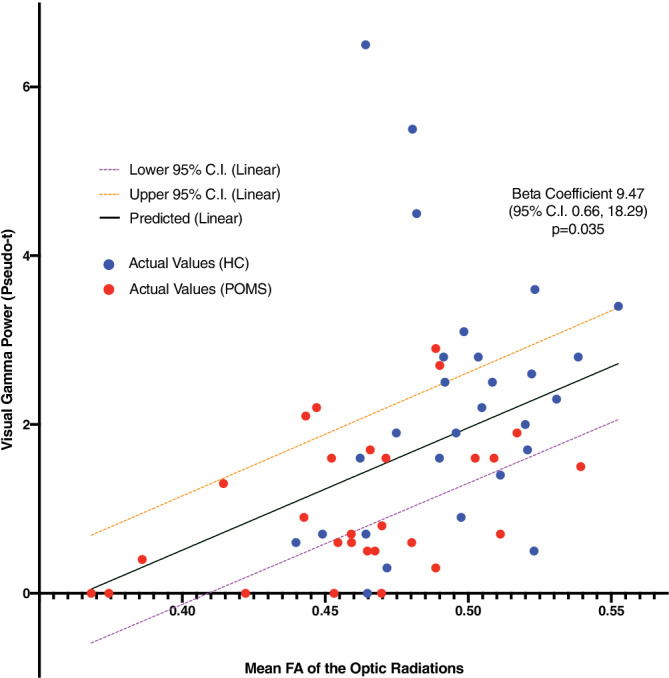
Fractional anisotropy (FA) of the optic radiations predicts visual gamma power (pseudo‐t) (generalized estimating equation [GEE] model, adjusted for case status (POMS vs. control) and age). POMS, pediatric‐onset multiple sclerosis; HC, healthy controls; CI, confidence interval. For each subject, FA and visual gamma power (pseudo‐t) values for the optic radiations and cuneus, respectively, in healthy controls (blue) and POMS participants (red) are depicted. This significant relationship (beta coefficient 9.47; 95% confidence interval 0.66, 18.29; *p* = .035) implicates a reduction in signaling via reduced white matter conduction in the optic radiations as a contributing factor toward reduced visual gamma power in POMS

The DTI sequences for one healthy control could not be analyzed due to motion artifact. As expected from our previous work, we detected a reduction in mean FA of the optic radiations in POMS compared to healthy controls (*p* < .001, Table 3). An increase in the mean FA of the optic radiations predicted an increase in visual gamma power (*p* = .001, Table 4, Figure 4). This relationship remained (*p* = .035, Table 4) after including disease status (POMS vs. control) as a covariate (beta coefficient for case vs. control status −0.85; 95% CI −1.54, −0.15, *p* = .017). Age was also included but did not impact the model (beta coefficient = .02; 95% CI −0.10, 0.142; *p* = .708).

**TABLE 4:** Structural–functional relationships

		Unadjusted models^a^		Adjusted models^b^	
Dependent variable	Explanatory variable(s)	Beta coefficient (95% CI)	*p*‐Value	Beta coefficient (95% CI)	*p*‐Value
Visual gamma power (pseudo‐t)	OR FA	14.50 (6.17, 22.82)	**.001**	9.47 (0.65, 18.29)	**.035**
	Normalized cuneus volume	528.90 (−548.76, 1,606.56)	.96	−94.51 (−1,161.70, 972.68)	.862
	Cuneus thickness (mm)	−0.68 (−2.97, 1.61)	.561	−1.49 (−3.61, 0.63)	.169
PMBR amplitude associated with peak latency (% change)	CST FA	137.84 (−124.56, 400.25)	.290	90.18 (−165.54, 345.89)	.473
	Normalized primary motor cortex volume	3,313.69 (−9,938.84, 16,566.22)	.612	−1888.08 (−14,687.48, 10,911.33)	.763
	Primary motor cortex thickness (mm)	0.93 (−22.82, 24.68)	.936	−7.51 (−29.95, 14.94)	.497
Reaction time (ms)	CST FA	−752.53 (−1,596.55, 91.48)	.078	−522.14 (−1,319.14, 274.85)	.188
	Normalized primary motor cortex volume	−42,162.56 (−82,059.53, −2,265.59)	**.039**	−28,400.64 (−66,638.03, 9,836.75)	.138
	Primary motor cortex thickness (mm)	−30.05 (−106.43, 46.32)	.426	0.12 (−70.68, 70.93)	.997

*Note:* All statistically significant values are provided in bold.

Abbreviations: CST, corticospinal tract; FA, fractional anisotropy; mm, millimeters; ms, milliseconds; OR, optic radiations; PMBR, postmovement beta rebound; POMS, pediatric‐onset multiple sclerosis.

^a^ Models include only the explanatory variables listed in the table.

^b^ Multivariate models, adjusted for disease status (POMS vs. control) and age.

### 3.4 Cuneus volume and cortical thickness

Cuneus thickness did not differ between POMS and controls (Table 3). However, normalized cuneus volume was decreased in POMS (*p* = .003, Table 3). Neither cuneus volume nor thickness predicted visual gamma power (Table 4).

### 3.5 Behavioral (motor) responses

After dropping trials for excessive head motion (>1 cm), noise (>10 pT), and anticipatory button‐press responses, an average of 85.6 (*SD* 10.6) trials for controls and 86.5 (*SD* 5.9) trials for POMS subjects were retained and included in the behavioral (motor) response and subsequent motor cortical oscillations analyses. The number of retained trials did not differ between controls and POMS subjects. Reaction time was delayed in POMS (mean 416.8 ms, *SD* 19.4) when compared to controls (mean 347.4 ms, *SD* 16.6, *p* = .018, Table 3).

### 3.6 Motor cortical oscillations

**FIGURE 5:**
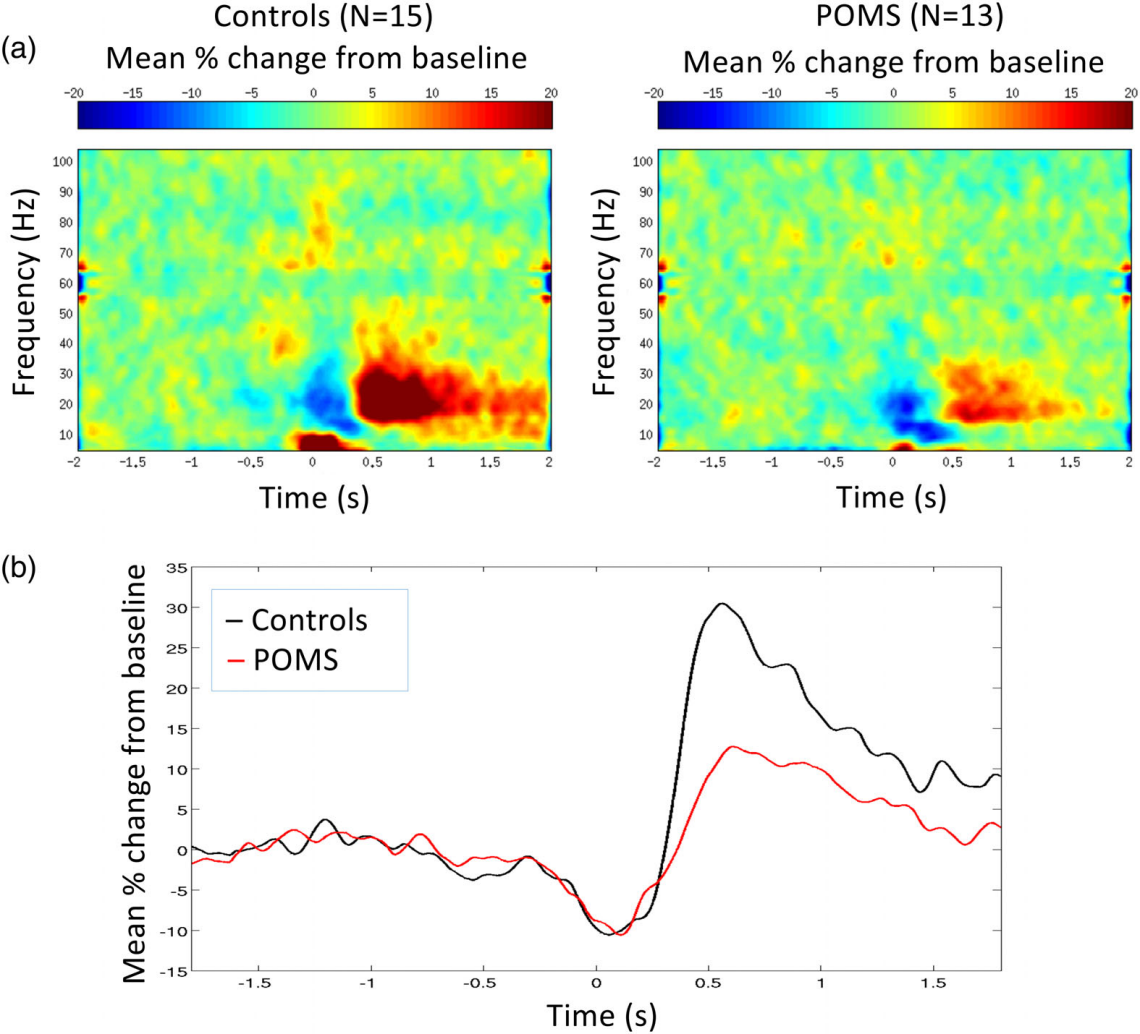
Postmovement beta rebound (PMBR) time‐frequency and mean amplitude at group level peak locations. POMS, pediatric‐onset multiple sclerosis; s, seconds. (a) Time–frequency plots demonstrate PMBR amplitude is reduced in POMS subjects at peak latency. (b) Mean PMBR peak amplitude, but not latency, is decreased in POMS (red) compared to healthy controls (black) (beta coefficient = −20.67; 95% confidence interval = −37.09, −4.24; *p* = .016)

The youngest (11‐year‐old) POMS subject produced a PMBR response (pseudo‐t) almost two standard deviations below the mean of the values for the other POMS subjects. Given our finding that subjects younger than 13 years of age do not typically produce a significant PMBR response (Gaetz et al., 2019), the 11‐year‐old POMS subject was dropped from the MEG motor analyses, as the absence of a robust PMBR could have been due to normal maturational factors. All peak MEG motor cortical oscillations were generated in the hemisphere of the brain contralateral (left) to the hand executing the button press (right). The group‐averaged differential SAM PMBR response observed following right finger button press is depicted for POMS subjects and controls in Figure 2b. PMBR time‐to‐peak latency, MRGS activity, and B‐ERD activity did not differ between POMS and controls (Table 3). However, the PMBR amplitude measured at peak latency was reduced in POMS (*p* = .011, Table 3, Figure 5).

### 3.7 DTI of the corticospinal tracts

Mean FA of the corticospinal tracts did not differ between groups (left hemisphere only, Table 3). Mean FA of the corticospinal tracts did not predict the PMBR amplitude measured at peak latency or reaction time (Table 4).

### 3.8 Primary motor cortex volume and cortical thickness

Neither normalized primary motor cortex volume nor thickness differed between POMS and healthy controls (left hemisphere only, Table 3). Neither measure predicted the PMBR amplitude measured at peak latency nor reaction time (Table 4).

## 4 DISCUSSION

We demonstrate differences in visual and motor cortical responses using MEG in POMS subjects compared to healthy youth. With respect to the visual cortex, visual gamma power is reduced in POMS, which appears to be related, at least in part, to the extent of disruption of visual pathway integrity and not to thickness or volume of the cuneus. Decreases in MEG oscillatory responses, structural measures, and reaction time occurred in the POMS subjects despite their young age, short disease duration (3.14 years), and low disability scores (median EDSS 1).

Our findings of reduced visual gamma band power in POMS are supported by prior studies in adult‐onset disease (Barratt et al., 2017; Stickland et al., 2019). In a cohort of 21 adult MS patients (mean age 42 years, *SD* 11) and 22 age‐ and sex‐matched healthy controls, a significant decrease in visual gamma band power occurred in adult MS subjects compared to controls (Barratt et al., 2017). Similarly, a reduction in gamma band power at an averaged peak amplitude in the time‐frequency domain was detected in 14 adult MS patients (mean age 43.5 years, *SD* 3.5) compared to 10 age‐ and sex‐matched healthy controls (Stickland et al., 2019).

While the neurobiological underpinnings of reduced gamma power have not been fully elucidated, cortical oscillations are generated by the harmonized firing of excitatory and inhibitory neurons. For example, regional decreases in the excitatory neurotransmitter glutamate have been demonstrated in primary progressive MS in adults and correlates in MS subjects with deficits in visuospatial learning and memory (Muhlert et al., 2014). Alternations in the inhibitory neurotransmitter, gamma‐aminobutyric acid (GABA), in the sensorimotor cortex in adult MS were associated with changes in motor performance (Bhattacharyya, Phillips, Stone, Bermel, & Lowe, 2013; Cawley et al., 2015), although the GABA concentrations varied in these studies. Relevant to our work, it has been postulated that disruptions in GABAergic signaling may contribute to decreased visual gamma power (Barratt et al., 2017; Stickland et al., 2019). In addition, GABA has been implicated in synaptic plasticity and neuroprotection (Saji & Reis, 1987; Stagg, 2014). Of note, the first clinical manifestations of POMS typically occur in adolescence during a time of active maturation and pruning of the cortex, thus we hypothesize that additional alternations in glutamate and GABA may be occurring unique to this patient population.

Reductions in cortical oscillatory activity may result from intracortical neurotransmitter alterations; however, MS is a complex disease with simultaneous gray and WM degenerative processes contributing to disability. Thus, we could not exclude the possibility of reduced cortical activity from anterograde degeneration of WM tracts. To our knowledge, no previous study in MS using MEG has specifically evaluated the relationship between gamma band power, volumetric, and DTI data. In our prior work, we demonstrated reductions in FA in normal‐appearing WM in the optic radiations of POMS subjects compared to healthy controls (Datta et al., 2019). This relationship remained after removing lesional tissue in the optic radiations. Thus, in the current paper, we explored the entirety of the optic radiations (lesional and nonlesional tissue), as this indeed represents the brain tissue operative for a given POMS patient. Decreases in FA predicted visual gamma power, even after accounting for group differences (POMS vs. control).

In adults with ON, DTI changes in the optic radiations were detected during the 12 months following a unilateral attack among 38 adults (19 of whom were confirmed to have MS during the study) compared to 23 healthy controls (Kolbe et al., 2016). Baseline FA of the optic radiations was reduced in adult MS subjects compared to controls, and the annualized rate of change after ON (−2.6% change in FA per year) was greater than in healthy controls (−0.51% per year, *p* = .006). Furthermore, changes in the FA of the optic radiations in these adult MS subjects were associated with the rate of change of V1 cortical thickness. In our prior cross‐sectional study, we demonstrated a reduction in visual cortex thickness in 20 POMS subjects compared to 22 age‐ and sex‐matched controls, as well as a relationship between FA of the optic radiations and visual cortex thinning (*p* = .017). However, V1, particularly the foveal confluence, was spared in POMS, while extrastriate cortical regions were preferentially affected.

In our current study, we specifically focused on the cuneus, the region of the cortex where the peak visual gamma response occurred (Barratt et al., 2017; Gaetz et al., 2012; Muthukumaraswamy et al., 2009). Cuneus volume was decreased in POMS compared to controls (*p* = .003), although groups did not differ in cuneus thickness. A relationship between cuneus volume and visual gamma power was not detected for either group. We acknowledge that, while the cuneus is the primary region responding to visual stimuli, other cortical regions and intercortical region communication likely contribute. Our small sample size precluded whole brain analytical methods, although such anamnestic methods will be a priority for future work.

Regarding oscillations in the primary motor cortex, the PMBR amplitude associated with peak latency was decreased in POMS compared to controls. We speculate that the reduction in PMBR amplitude suggests reduced neuronal firing, which we hypothesize relates to either reduced neuronal density or decreased neuronal excitability. However, unlike the visual system, differences in PMBR amplitude could not be explained in the POMS group by variations in corticospinal tract FA or primary motor cortex volume or thickness. Primary motor cortex thickness and volume were not statistically different between groups, although both were decreased in the POMS group. Adult MS patients have a delay in the time‐to‐peak of the PMBR response (Barratt et al., 2017); however, PMBR time‐to‐peak latency in POMS was not impacted.

While we did not observe a relationship between PMBR time‐to‐peak latency and primary motor cortex structural measures, we did observe a negative relationship between reaction time and primary motor cortex volume, although this relationship was not significant after accounting for group differences (POMS vs. controls) and age. Thinning of the primary motor cortex relates to performance on a timed motor task in POMS (Datta et al., 2016).

We acknowledge the following limitations. POMS is a rare disorder, limiting recruitment for research studies. We further recognize the large variance on the estimates in this small sample size. Although we did not detect a relationship between ON history and visual gamma power, we acknowledge that our small cohort of POMS subjects (only half of whom had a history of ON) may have been underpowered to appropriately investigate the effects of ON history on the imaging metrics. We did not detect significant structural differences in the motor pathways, although a larger sample size is needed to further corroborate these findings. The projector was replaced near the end of the study while age‐matched controls were being enrolled, although we did not detect a difference in visual gamma power before and after the change.

We have demonstrated decreases in visual gamma power, cuneus volume, and mean FA of the optic radiations in POMS subjects despite their young age, mild disability, and short disease duration. This supports our hypothesis of an early vulnerability of the extrastriate visual system in POMS. Further research is needed to determine the relationship between these findings and the patients' higher order visual and motor performance. Specifically, network connectivity analysis would be of value to define response patterns and whether reduced cortical oscillations are associated with a predicted increase and expansion of regional activation to sustain neurological performance and perception.

## CONFLICTS OF INTEREST

A. W. has received research support from the NIH (NINDS K23NS069806; PI; R01NS071463, site investigator), Biogen Idec (PI), IONIS Pharmaceuticals (PI), United Leukodystrophy Foundation, and the Children's Hospital of Philadelphia (Foerderer Award, 2016, PI), royalties from UpToDate, and served as a consultant to Optum. Geraldine Liu discloses her spouse's royalties for Liu, Volpe, Galetta: Neuro‐Ophthalmology, Diagnosis and Management, 2010, Elsevier. B. L. B. serves as an advisor to Novartis. She also serves as a nonremunerated advisor to Biogen, Sanofi, and Teva Neuroscience. All the other authors declare no conflicts of interest.

## Data Availability

Research data are not shared.

## References

[hbm25126-bib-0001] Adjamian, P. , Holliday, I. E. , Barnes, G. R. , Hillebrand, A. , Hadjipapas, A. , & Singh, K. D. (2004). Induced visual illusions and gamma oscillations in human primary visual cortex. European Journal of Neuroscience, 20(2), 587–592. 10.1111/j.1460-9568.2004.03495.x 15233769

[hbm25126-bib-0002] Andersson, J. L. R. , Jenkinson, M. , & Smith, S. (2007). Non‐linear registration aka spatial normalisation FMRIB Technical Report TR07JA2. Retrieved from https://www.fmrib.ox.ac.uk/datasets/techrep/tr07ja2/tr07ja2.pdf

[hbm25126-bib-0052] Arpin, D. J. , Heinrichs‐Graham, E. , Gehringer, J. E. , Zabad, R. , Wilson, T. W. , & Kurz, M. J. (2017). Altered sensorimotor cortical oscillations in individuals with multiple sclerosis suggests a faulty internal Model. Human Brain Mapping, 38(8), 4009–4018. 10.1002/hbm.23644 28485884 PMC6867014

[hbm25126-bib-0003] Balk, L. , Steenwijk, M. , Tewarie, P. , Daams, M. , Killestein, J. , Wattjes, M. , … Petzold, A. (2015). Bidirectional trans‐synaptic axonal degeneration in the visual pathway in multiple sclerosis. Journal of Neurology, Neurosurgery, and Psychiatry, 86(4), 419–424. 10.1136/jnnp-2014-308189 24973342

[hbm25126-bib-0004] Barratt, E. L. , Tewarie, P. K. , Clarke, M. A. , Hall, E. L. , Gowland, P. A. , Morris, P. G. , … Brookes, M. J. (2017). Abnormal task driven neural oscillations in multiple sclerosis: A visuomotor MEG study. Human Brain Mapping, 38(5), 2441–2453. 10.1002/hbm.23531 28240392 PMC6866959

[hbm25126-bib-0005] Bhattacharyya, P. K. , Phillips, M. D. , Stone, L. A. , Bermel, R. A. , & Lowe, M. J. (2013). Sensorimotor cortex gamma‐aminobutyric acid concentration correlates with impaired performance in patients with MS. American Journal of Neuroradiology, 34(9), 1733–1739. 10.3174/ajnr.A3483 23493890 PMC7965622

[hbm25126-bib-0006] Cawley, N. , Solanky, B. S. , Muhlert, N. , Tur, C. , Edden, R. A. E. , Wheeler‐Kingshott, C. A. M. , … Ciccarelli, O. (2015). Reduced gamma‐aminobutyric acid concentration is associated with physical disability in progressive multiple sclerosis. Brain, 138(Pt 9), 2584–2595. 10.1093/brain/awv209 26304151 PMC4643627

[hbm25126-bib-0007] Cheyne, D. , Bells, S. , Ferrari, P. , Gaetz, W. , & Bostan, A. C. (2008). Self‐paced movements induce high‐frequency gamma oscillations in primary motor cortex. NeuroImage, 42(1), 332–342. 10.1016/j.neuroimage.2008.04.178 18511304

[hbm25126-bib-0008] Cheyne, D. , & Ferrari, P. (2013). MEG studies of motor cortex gamma oscillations: Evidence for a gamma “fingerprint” in the brain? Frontiers in Human Neuroscience, 7(Sep), 575 10.3389/fnhum.2013.00575 24062675 PMC3774986

[hbm25126-bib-0053] Datta, R. , Till, C. , De Somma, E. , Akbar, N. , Lysenko, M. , Yeh, A. E. , … Banwell, B. (2016). Cortical mantle thinning in pediatric MS: correlations with motor function. ECTRIMS Online Library.

[hbm25126-bib-0009] Datta, R. , Sollee, J. R. , Lavery, A. M. , Ficerai‐Garland, G. , Karoscik, K. , Liu, G. , … Waldman, A. T. (2019). Effects of optic neuritis, T2 lesions, and microstructural diffusion integrity in the visual pathway on cortical thickness in pediatric‐onset multiple sclerosis. Journal of Neuroimaging, 29(6), 760–770. 10.1111/jon.12654 31317617 PMC10637320

[hbm25126-bib-0010] Desikan, R. S. , Ségonne, F. , Fischl, B. , Quinn, B. T. , Dickerson, B. C. , Blacker, D. , … Killiany, R. J. (2006). An automated labeling system for subdividing the human cerebral cortex on MRI scans into gyral based regions of interest. NeuroImage, 31(3), 968–980. 10.1016/j.neuroimage.2006.01.021 16530430

[hbm25126-bib-0011] Eickhoff, S. B. , Stephan, K. E. , Mohlberg, H. , Grefkes, C. , Fink, G. R. , Amunts, K. , & Zilles, K. (2005). A new SPM toolbox for combining probabilistic cytoarchitectonic maps and functional imaging data. NeuroImage, 25(4), 1325–1335. 10.1016/j.neuroimage.2004.12.034 15850749

[hbm25126-bib-0012] Fries, P. , Reynolds, J. H. , Rorie, A. E. , & Desimone, R. (2001). Modulation of oscillatory neuronal synchronization by selective visual attention. Science, 291(5508), 1560–1563. 10.1126/science.1055465 11222864

[hbm25126-bib-0013] Fries, P. , Schröder, J. H. , Roelfsema, P. R. , Singer, W. , & Engel, A. K. (2002). Oscillatory neuronal synchronization in primary visual cortex as a correlate of stimulus selection. Journal of Neuroscience, 22(9), 3739–3754. 10.1523/jneurosci.22-09-03739.2002 11978850 PMC6758402

[hbm25126-bib-0014] Gabilondo, I. , Martínez‐Lapiscina, E. H. , Martínez‐Heras, E. , Fraga‐Pumar, E. , Llufriu, S. , Ortiz, S. , … Villoslada, P. (2014). Trans‐synaptic axonal degeneration in the visual pathway in multiple sclerosis. Annals of Neurology, 75(1), 98–107. 10.1002/ana.24030 24114885

[hbm25126-bib-0015] Gaetz, W. , Edgar, J. C. , Wang, D. J. , & Roberts, T. P. L. (2011). Relating MEG measured motor cortical oscillations to resting gamma‐aminobutyric acid (GABA) concentration. NeuroImage, 55(2), 616–621. 10.1016/j.neuroimage.2010.12.077 21215806 PMC3411117

[hbm25126-bib-0016] Gaetz, W. , Rhodes, E. , Bloy, L. , Blaskey, L. , Jackel, C. R. , Brodkin, E. S. , … Roberts, T. P. L. (2019). Evaluating motor cortical oscillations and age‐related change in autism spectrum disorder. NeuroImage, 207, 116349 10.1016/j.neuroimage.2019.116349 31726253

[hbm25126-bib-0017] Gaetz, W. , Roberts, T. P. L. L. , Singh, K. D. , & Muthukumaraswamy, S. D. (2012). Functional and structural correlates of the aging brain: Relating visual cortex (V1) gamma band responses to age‐related structural change. Human Brain Mapping, 33(9), 2035–2046. 10.1002/hbm.21339 21769992 PMC3197906

[hbm25126-bib-0018] Gorman, M. P. , Healy, B. C. , Polgar‐Turcsanyi, M. , & Chitnis, T. (2009). Increased relapse rate in pediatric‐onset compared with adult‐onset multiple sclerosis. Archives of Neurology, 66(1), 54–59. 10.1001/archneurol.2008.505 19139299

[hbm25126-bib-0019] Graves, J. S. , Chohan, H. , Cedars, B. , Arnow, S. , Yiu, H. , Waubant, E. , & Green, A. (2017). Sex differences and subclinical retinal injury in pediatric‐onset MS. Multiple Sclerosis (Houndmills, Basingstoke, England), 23(3), 447–455. 10.1177/1352458516652497 27306618

[hbm25126-bib-0020] Guo, C. , Ferreira, D. , Fink, K. , Westman, E. , & Granberg, T. (2019). Repeatability and reproducibility of FreeSurfer, FSL‐SIENAX and SPM brain volumetric measurements and the effect of lesion filling in multiple sclerosis. European Radiology, 29(3), 1355–1364. 10.1007/s00330-018-5710-x 30242503 PMC6510869

[hbm25126-bib-0021] Hall, S. D. , Holliday, I. E. , Hillebrand, A. , Furlong, P. L. , Singh, K. D. , & Barnes, G. R. (2005). Distinct contrast response functions in striate and extra‐striate regions of visual cortex revealed with magnetoencephalography (MEG). Clinical Neurophysiology, 116(7), 1716–1722. 10.1016/j.clinph.2005.02.027 15953561

[hbm25126-bib-0022] Hämäläinen, M. , Hari, R. , Ilmoniemi, R. J. , Knuutila, J. , & Lounasmaa, O. V. (1993). Magnetoencephalography theory, instrumentation, and applications to noninvasive studies of the working human brain. Reviews of Modern Physics, 65(2), 413–497. 10.1103/RevModPhys.65.413

[hbm25126-bib-0023] Henrie, J. A. , & Shapley, R. (2005). LFP power spectra in V1 cortex: The graded effect of stimulus contrast. Journal of Neurophysiology, 94(1), 479–490. 10.1152/jn.00919.2004 15703230

[hbm25126-bib-0024] Jensen, O. , Kaiser, J. , & Lachaux, J. P. (2007). Human gamma‐frequency oscillations associated with attention and memory. Trends in Neurosciences, 30(7), 317–324. 10.1016/j.tins.2007.05.001 17499860

[hbm25126-bib-0025] Kolasinski, J. , Stagg, C. J. , Chance, S. A. , DeLuca, G. C. , Esiri, M. M. , Chang, E. H. , … Johansen‐Berg, H. (2012). A combined post‐mortem magnetic resonance imaging and quantitative histological study of multiple sclerosis pathology. Brain, 135(10), 2938–2951. 10.1093/brain/aws242 23065787 PMC3470716

[hbm25126-bib-0026] Kolbe, S. C. , van der Walt, A. , Butzkueven, H. , Klistorner, A. , Egan, G. F. , & Kilpatrick, T. J. (2016). Serial diffusion tensor imaging of the optic radiations after acute optic neuritis. Journal of Ophthalmology, 2016, 2764538 10.1155/2016/2764538 27555964 PMC4983385

[hbm25126-bib-0027] Little, S. , Bonaiuto, J. , Barnes, G. , & Bestmann, S. (2019). Human motor cortical beta bursts relate to movement planning and response errors. PLoS Biology, 17(10), e3000479 10.1371/journal.pbio.3000479 31584933 PMC6795457

[hbm25126-bib-0028] Marrie, R. A. , Cohen, J. , Stuve, O. , Trojano, M. , Sørensen, P. S. , Reingold, S. , … Reider, N. (2015). A systematic review of the incidence and prevalence of comorbidity in multiple sclerosis: Overview. Multiple Sclerosis Journal, 21(3), 263–281. 10.1177/1352458514564491 25623244 PMC4361468

[hbm25126-bib-0029] Melloni, L. , Molina, C. , Pena, M. , Torres, D. , Singer, W. , & Rodriguez, E. (2007). Synchronization of neural activity across cortical areas correlates with conscious perception. Journal of Neuroscience, 27(11), 2858–2865. 10.1523/JNEUROSCI.4623-06.2007 17360907 PMC6672558

[hbm25126-bib-0030] Muhlert, N. , Atzori, M. , de Vita, E. , Thomas, D. L. , Samson, R. S. , Wheeler‐Kingshott, C. A. M. , … Ciccarelli, O. (2014). Memory in multiple sclerosis is linked to glutamate concentration in grey matter regions. Journal of Neurology, Neurosurgery and Psychiatry, 85(8), 834–840. 10.1136/jnnp-2013-306662 PMC4112488 24431465

[hbm25126-bib-0031] Muthukumaraswamy, S. D. , Edden, R. A. E. , Jones, D. K. , Swettenham, J. B. , & Singh, K. D. (2009). Resting GABA concentration predicts peak gamma frequency and fMRI amplitude in response to visual stimulation in humans. Proceedings of the National Academy of Sciences of the United States of America, 106(20), 8356–8361. 10.1073/pnas.0900728106 19416820 PMC2688873

[hbm25126-bib-0032] Muthukumaraswamy, S. D. , & Singh, K. D. (2013). Visual gamma oscillations: The effects of stimulus type, visual field coverage and stimulus motion on MEG and EEG recordings. NeuroImage, 69, 223–230. 10.1016/j.neuroimage.2012.12.038 23274186

[hbm25126-bib-0033] Muthukumaraswamy, S. D. (2010). Functional properties of human primary motor cortex gamma oscillations. Journal of Neurophysiology, 104(5), 2873–2885. 10.1152/jn.00607.2010 20884762

[hbm25126-bib-0034] Muthukumaraswamy, S. D. , & Singh, K. D. (2009). Functional decoupling of BOLD and gamma‐band amplitudes in human primary visual cortex. Human Brain Mapping, 30(7), 2000–2007. 10.1002/hbm.20644 18729078 PMC6870698

[hbm25126-bib-0035] Muthukumaraswamy, S. D. , Singh, K. D. , Swettenham, J. B. , & Jones, D. K. (2010). Visual gamma oscillations and evoked responses: Variability, repeatability and structural MRI correlates. NeuroImage, 49(4), 3349–3357. 10.1016/j.neuroimage.2009.11.045 19944770

[hbm25126-bib-0036] Nichols, T. E. , & Holmes, A. P. (2002). Nonparametric permutation tests for functional neuroimaging: A primer with examples. Human Brain Mapping, 15(1), 1–25. 10.1002/hbm.1058 11747097 PMC6871862

[hbm25126-bib-0037] Obenaus, A. , Yong‐Hing, C. J. , Tong, K. A. , & Sarty, G. E. (2001). A reliable method for measurement and normalization of pediatric hippocampal volumes. Pediatric Research, 50(1 I), 124–132. 10.1203/00006450-200107000-00022 11420429

[hbm25126-bib-0038] Parra, J. , Kalitzin, S. N. , Iriarte, J. , Blanes, W. , Velis, D. N. , & Lopes da Silva, F. H. (2003). Gamma‐band phase clustering and photosensitivity: Is there an underlying mechanism common to photosensitive epilepsy and visual perception? Brain, 126(5), 1164–1172. 10.1093/brain/awg109 12690055

[hbm25126-bib-0039] Saji, M. , & Reis, D. J. (1987). Delayed transneuronal death of substantia nigra neurons prevented by γ‐aminobutyric acid agonist. Science, 235(4784), 66–69. 10.1126/science.3798095 3798095

[hbm25126-bib-0040] Sanfilipo, M. P. , Benedict, R. H. B. , Zivadinov, R. , & Bakshi, R. (2004). Correction for intracranial volume in analysis of whole brain atrophy in multiple sclerosis: The proportion vs. residual method. NeuroImage, 22(4), 1732–1743. 10.1016/j.neuroimage.2004.03.037 15275929

[hbm25126-bib-0041] Schmidt, P. , Gaser, C. , Arsic, M. , Buck, D. , Förschler, A. , Berthele, A. , … Mühlau, M. (2012). An automated tool for detection of FLAIR‐hyperintense white‐matter lesions in multiple sclerosis. NeuroImage, 59(4), 3774–3783. 10.1016/j.neuroimage.2011.11.032 22119648

[hbm25126-bib-0042] Simone, I. L. , Carrara, D. , Tortorella, C. , Liguori, M. , Lepore, V. , Pellegrini, F. , … Livrea, P. (2002). Course and prognosis in early‐onset MS: Comparison with adult‐onset forms. Neurology, 59(12), 1922–1928. 10.1212/01.WNL.0000036907.37650.8E 12499484

[hbm25126-bib-0043] Smith, S. M. , Jenkinson, M. , Johansen‐Berg, H. , Rueckert, D. , Nichols, T. E. , Mackay, C. E. , … Behrens, T. E. J. (2006). Tract‐based spatial statistics: Voxelwise analysis of multi‐subject diffusion data. NeuroImage, 31(4), 1487–1505. 10.1016/j.neuroimage.2006.02.024 16624579

[hbm25126-bib-0044] Stagg, C. J. (2014). Magnetic resonance spectroscopy as a tool to study the role of GABA in motor‐cortical plasticity. NeuroImage, 86, 19–27. 10.1016/j.neuroimage.2013.01.009 23333699

[hbm25126-bib-0045] Stickland, R. , Allen, M. , Magazzini, L. , Singh, K. D. , Wise, R. G. , & Tomassini, V. (2019). Neurovascular coupling during visual stimulation in multiple sclerosis: A MEG‐fMRI study. Neuroscience, 403, 54–69. 10.1016/j.neuroscience.2018.03.018 29580963 PMC6458991

[hbm25126-bib-0046] Thompson, A. J. , Banwell, B. L. , Barkhof, F. , Carroll, W. M. , Coetzee, T. , Comi, G. , … Cohen, J. A. (2018). Diagnosis of multiple sclerosis: 2017 revisions of the McDonald criteria. The Lancet Neurology, 17(2), 162–173. 10.1016/S1474-4422(17)30470-2 29275977

[hbm25126-bib-0047] Voevodskaya, O. (2014). The effects of intracranial volume adjustment approaches on multiple regional MRI volumes in healthy aging and Alzheimer's disease. Frontiers in Aging Neuroscience, 6, 264 10.3389/fnagi.2014.00264 25339897 PMC4188138

[hbm25126-bib-0048] Waldman, A. , Ghezzi, A. , Bar‐Or, A. , Mikaeloff, Y. , Tardieu, M. , & Banwell, B. (2014). Multiple sclerosis in children: An update on clinical diagnosis, therapeutic strategies, and research. The Lancet Neurology, 13(9), 936–948. 10.1016/S1474-4422(14)70093-6 25142460 PMC4443918

[hbm25126-bib-0049] Waldman, A. T. , Hiremath, G. , Avery, R. A. , Conger, A. , Pineles, S. L. , Loguidice, M. J. , … Calabresi, P. A. (2014). Monocular and binocular low‐contrast visual acuity and optical coherence tomography in pediatric multiple sclerosis. Multiple Sclerosis and Related Disorders, 3(3), 326–334. 10.1016/j.msard.2013.10.008 PMC3964624 24683535

[hbm25126-bib-0050] Waldman, A. T. , Liu, G. T. , Lavery, A. M. , Liu, G. , Gaetz, W. , Aleman, T. S. , & Banwell, B. L. (2017). Optical coherence tomography and visual evoked potentials in pediatric MS. Neurology ‐ Neuroimmunology Neuroinflammation, 4(4), e356 10.1212/NXI.0000000000000356 28626779 PMC5459791

[hbm25126-bib-0051] Yeh, E. A. , Weinstock‐Guttman, B. , Lincoff, N. , Reynolds, J. , Weinstock, A. , Madurai, N. , … Ramanathan, M. (2009). Retinal nerve fiber thickness in inflammatory demyelinating diseases of childhood onset. Multiple Sclerosis (Houndmills, Basingstoke, England), 15(7), 802–810. 10.1177/1352458509104586 19465453

